# The Role of Interleukin-10 and Hyaluronan in Murine Fetal Fibroblast Function *In Vitro*: Implications for Recapitulating Fetal Regenerative Wound Healing

**DOI:** 10.1371/journal.pone.0124302

**Published:** 2015-05-07

**Authors:** Swathi Balaji, Alice King, Emily Marsh, Maria LeSaint, Sukanta S. Bhattacharya, Nathaniel Han, Yashu Dhamija, Rajeev Ranjan, Louis D. Le, Paul L. Bollyky, Timothy M. Crombleholme, Sundeep G. Keswani

**Affiliations:** 1 Laboratory for Regenerative Wound Healing, Division of Pediatric, General, Thoracic and Fetal Surgery, Cincinnati Children's Hospital Medical Center, Cincinnati, Ohio, United States of America; 2 Division of Infectious Diseases, Department of Medicine, Stanford University School of Medicine, Palo Alto, California, United States of America; 3 Center for Children's Surgery, Children’s Hospital Colorado and The University of Colorado School of Medicine, Aurora, Colorado, United States of America; 4 Division of Pediatric General and Thoracic Surgery, Baylor College of Medicine and Texas Children's Hospital, Houston, Texas, United States of America; National University of Singapore, SINGAPORE

## Abstract

**Background:**

Mid-gestation fetal cutaneous wounds heal scarlessly and this has been attributed in part to abundant hyaluronan (HA) in the extracellular matrix (ECM) and a unique fibroblast phenotype. We recently reported a novel role for interleukin 10 (IL-10) as a regulator of HA synthesis in the fetal ECM, as well as the ability of the fetal fibroblast to produce an HA-rich pericellular matrix (PCM). We hypothesized that IL-10-mediated HA synthesis was essential to the fetal fibroblast functional phenotype and, moreover, that this phenotype could be recapitulated in adult fibroblasts via supplementation with IL-10 via an HA dependent process.

**Methodology/Principal Findings:**

To evaluate the differences in functional profile, we compared metabolism (MTS assay), apoptosis (caspase-3 staining), migration (scratch wound assay) and invasion (transwell assay) between C57Bl/6J murine fetal (E14.5) and adult (8 weeks) fibroblasts. We found that fetal fibroblasts have lower rates of metabolism and apoptosis, and an increased ability to migrate and invade compared to adult fibroblasts, and that these effects were dependent on IL-10 and HA synthase activity. Further, addition of IL-10 to adult fibroblasts resulted in increased fibroblast migration and invasion and recapitulated the fetal phenotype in an HA-dependent manner.

**Conclusions/Significance:**

Our data demonstrates the functional differences between fetal and adult fibroblasts, and that IL-10 mediated HA synthesis is essential for the fetal fibroblasts' enhanced invasion and migration properties. Moreover, IL-10 via an HA-dependent mechanism can recapitulate this aspect of the fetal phenotype in adult fibroblasts, suggesting a novel mechanism of IL-10 in regenerative wound healing.

## Introduction

Cutaneous wound repair occurs in a highly orchestrated sequence of events that begins with hemostasis, proceeds to inflammation and proliferation, and concludes with dermal remodeling. In most postnatal mammals, this process results in the formation of a scar [1]. In contrast, the mid-gestation mammalian fetus is capable of healing dermal wounds without scar formation and includes the reconstitution of dermal appendages, which results in wound repair indistinguishable from the surrounding uninjured skin [1–5]. Although the distinct healing properties of fetal wounds have been known for over thirty years, the complete underlying mechanisms of fetal regenerative healing still remain poorly understood [6]. Previous studies have demonstrated that fetal wounds have an attenuated inflammatory response [7–11], and are composed of an extracellular matrix (ECM) with an abundance of the glycosaminoglycan, hyaluronan (HA) [12–17].

The synthesis and remodeling of the ECM is primarily regulated by dermal fetal fibroblast (FFB) and it is believed to be an integral contributor to the fetal regenerative phenotype [18]. Fibroblasts synthesize and respond to numerous growth factors and extracellular matrix components, which stimulate and permit cellular proliferation and migration. The migration and proliferation of fibroblasts into the acute postnatal wound is signaled by potent tissue growth factors including platelet-derived growth factor (PDGF), transforming growth factor (TGF-β), and basic fibroblast growth factor (bFGF) [19]. After injury, the wound fibroblast number increases via migration from adjacent unwounded tissue, but soon after, fibroblast proliferation rapidly expand the total pool of fibroblasts. Fibroblasts require a scaffold or matrix to bind to and move across to enter the acute wound environment and initiate tissue repair. Previous reports have described functional differences between adult fibroblasts (AFB) and fetal fibroblasts (FFB) including differences in migratory phenotype [20–22], proliferation [23], differentiation and cytokine/ ECM synthesis [22, 24, 25] [26]. Further phenotypic differences between FFB and AFB have not been fully elucidated.

FFB and AFB also differ in their production of hyaluronic acid (HA). HA promotes cell migration and proliferation early in the repair process. As cells migrate into the wound, they secrete hyaluronidase and plasminogen activator to degrade the HA and fibrin. As a result, fibroblast migration is inhibited and fibroblast differentiation and mature connective tissue synthesis is induced [27]. While both fetal and postnatal skin respond by producing HA following injury, the fetus is characterized by elevated and prolonged levels of high molecular weight hyaluronic acid (HMW-HA) [28, 29]. Compared to AFB, FFB produce more HA and express higher levels of CD44, the main HA receptor [15]. The high levels of HA in the fetal ECM may provide a permissive hydrated environment for fibroblasts to migrate through the wound efficiently, thereby attenuating scar formation. It has been suggested that the superior migration ability of the FFB may be a direct result of this unique HA-rich extracellular matrix [24, 30].

Our laboratory has previously reported an essential role of the anti-inflammatory cytokine IL-10 in the fetal regenerative phenotype. Fetal skin has been shown to have higher levels of IL-10 [31] and fetal wounds in transgenic *IL-10* ^*-/-*^ mice heal with a scar at a gestational age that should heal scarlessly [32]. Most convincingly, multiple studies have demonstrated the ability of IL-10 overexpression to induce scarless healing in postnatal wounds in both animal and human clinical trials [33–35]. The mechanism of the regenerative effects of IL-10 is considered to be an attenuation of the inflammatory response. However, we have recently reported an additional mechanism for IL-10, beyond its immunoregulatory role, as a regulator of HA synthesis in the fetal extracellular matrix as well as the regulator of FFB’s ability to produce an HA-rich PCM [36–38]. The role of IL-10 in the functional capabilities of the FFB and the ability of IL-10 to recapitulate the fetal phenotype in AFB is unknown.

Taken together, we hypothesize that IL-10 mediated HA synthesis is essential to the FFB functional profile and that recapitulation of this function in AFB can be achieved by the addition of IL-10. To test this hypothesis, we first defined the functional profile of FFB and AFB by assaying cellular metabolism, apoptosis, migration and invasion. We then determined which functional parameters are IL-10 and HA dependent through a series of loss-of-function experiments using transgenic *IL-10* ^*-/-*^ fibroblasts and a novel inhibitor of HA synthesis, 4-methylumbelliferone (4-MU). 4-MU is a well-established inhibitor of HA synthesis [39]. 4-MU acts as a competitive substrate for UDP-glucuronyltransferase (UGT), an enzyme that converts Glucosamine to UDP-Glucosamine [40–42]. We and others have previously demonstrated that 4-MU inhibits HA production by other cell types without increasing cell death [43]. Lastly, we investigated the role of IL-10 in postnatal fibroblast function by gain-of-function experiments using the addition of recombinant IL-10 (rIL-10).

## Methods

### Cell culture

All protocols were approved by the Cincinnati Children’s Hospital Institutional Animal Care and Use Committee. Primary fetal murine dermal fibroblasts were isolated from mid-gestation age fetuses (day 14.5) from control C57BL/6J and transgenic *IL-10* ^*-/-*^ mice (Strain numbers 000664 and 002251 respectively, Jackson Laboratories, Bar Harbor, ME) using standard isolation protocols [44]. Primary adult murine dermal fibroblasts were obtained from control C57BL/6J adult mice (8–12 weeks). Only dorsal skin between the forelimbs and hind limbs was excised and used for cell isolation. Cells were plated in BD Falcone cell culture flasks (BD Biosciences, Bedford, MA) without any additional surface coating, and the fibroblast culture was maintained in Dulbecco’s Modified Eagle’s media (DMEM) (GIBCO, Carlsbad, CA) supplemented with 10% bovine growth serum (BGS) (Hyclone, Logan, UT), 1% sodium pyruvate, penicillin 100 units, streptomycin 100 μg and amphotericin B 0.25 μg (PSF) (Invitrogen, Carlsbad, CA), and maintained in a humidified chamber at 37°C with 5% CO_2_. Cells between passages 5–10 were used for experiments.

### Metabolic Assay

Cell metabolic activity was evaluated using the MTS (3-(4, 5-dimethylthiazol-2-yl)-5-(3-carboxymethoxyphenyl)-2-(4-sulfophenyl)-2H-tetrazolium) assay (Roche, Indianapolis, IN). This is a colorimetric assay which measures cellular metabolic activity through the reduction of tetrazolium dye, MTS, to formazan via NAD(P)H-dependent cellular oxidoreductase enzymes [45]. Independent baseline viability curves were generated for fetal C57BL/6J, fetal *IL-10* ^*-/-*^ and adult C57BL/6J fibroblast lines to account for variances in mitochondrial dehydrogenase activity between cell types. Cells were seeded in 96-well plates at a density of 1000, 2000, 3000, 4000, 5000, 7500, 10000 and 15000 cells per well in DMEM with 10% BGS and PSF, and incubated for 4 hours at 37°C and 5% CO_2_ to allow fibroblasts to settle and attach. The relative number of viable cells was determined after 4 hours by the addition of 200 μl MTS solution and absorbance read at 490 nm after 2 hours incubation (S1 Fig). All the experimental groups were evaluated at 48 hours. Cells were seeded in a 96-well plate at a density of 5000 cells per well in DMEM with 10% BGS and PSF with or without the treatments (IL-10 (200 ng/ml; PeproTech, Rocky Hill, NJ) and/or 4-MU (0.3 mM/ml; Sigma-Aldrich Corp. St. Louis, MO). The dose of IL-10 was determined by performing a dose escalation study using various IL-10 levels and examining the effect on HA synthase 1 (HAS1) gene expression. 200ng/ml of IL-10 represented the plateau of the maximal increase in HAS1 gene expression. The dose of 4-MU was chosen based on previous reports of levels between 0.25–1.5 mM [46–50] achieving efficient and effective inhibition of HA synthesis. While higher levels of 4-MU may have a more pronounced effect on inhibiting HA synthesis in our system, we choose to use a lower dose to reduce any toxicity. After 48 hours of incubation, 200 μl MTS solution was added and incubated for another 2 hours. Absorbance was measured at 490 nm. Cellular activity for each experimental group was calculated using respective baseline standard curves for each cell type in question. The assay was performed in triplicates for each experimental group with cells at passage 5 or 6 from three independent isolations (n = 3).

### Cell Apoptosis

FFB and AFB were plated at 7500 cells per well in cell culture chamber slides in DMEM containing 10% BGS and PSF. The chamber slide surfaces were not treated with any additional coating. Cells were allowed to attach overnight to the slides followed by aspiration of the media and unattached cells. Fresh DMEM containing 2% BGS and PSF was added to the cultures with or without IL-10 (200 ng/ml) and/or 4-MU (0.3 mM/ml)). Cells were incubated for 48 hours, after which the chamber slides were washed with PBS and cells were fixed in 4% paraformaldehyde for 15 minutes at room temperature. Immunocytochemistry for apoptosis was performed. Endogenous peroxidase activity was blocked with 3% hydrogen peroxide for 10 min at room temperature. Non-specific protein binding was blocked with 5% normal goat serum in phosphate buffered saline plus 0.1% Tween-20 (PBSTw). Anti-cleaved caspase-3 (1:400, Cell Signaling Technology, Danvers, MA) antibody was incubated for 2 hours at room temperature. Following washes in PBS, slides were incubated in goat anti-rabbit IgG secondary antibody conjugated with HRP (1:200, Vector Laboratories, Burlingame, CA) for 15 minutes at room temperature. Slides were washed and developed in DAB and counter stained with nuclear fast red stain, cleared and mounted. Cells were imaged with bright field microscopy using a Nikon 80i microscope and Nikon Elements Software, v3.2 (Nikon Instruments, Melville, NY). The rate of apoptosis was calculated as the number of caspase-3 positive cells over the total number of cells per high-powered field (20X) averaged over ten images. Immunohistochemistry was performed in triplicate for each experimental group with cells at passage 5 or 6 from three independent isolations (n = 3).

### Migration Assay

FFB and AFB were seeded in 12-well cell culture plates without any additional surface coating at a density of 1.0x10^6^ cells per well in 3 ml DMEM containing 10% BGS and allowed to adhere overnight. The media was removed and cells were then serum starved in DMEM containing only 2% BGS for 24 hours prior to the treatment. After this time, treatment and control groups were established with or without the supplementation of IL-10 (200 ng/ml) and/or 4-MU (0.3 mM/ml) to the conditioned media. A scratch defect was created in the cell monolayer along the diameter in each group using a sterile pipette tip (20–200 μl tip). Four points were marked and defined along the scratch defect, which were used as reference points to capture photographic images to trace defect closure at multiple time points, including immediately following scratching (0 hours), at 6 hours, 12 hours, and after 24 hours incubation. Phase contrast imaging with an Axiovert 100M inverted microscope (Carl Zeiss, Thornwood, NY) was used to obtain 4x images. The unfilled scratch defect area was measured at each reference point per well, averaged over 4 fields per well (S2 Fig). Data was presented as extent of wound closure, that is, the percentage by which the scratch area has decreased at a given time point for each treatment as compared to the original defect (at 0 hours). All experiments were carried out in triplicate with cells from three independent isolations (n = 3) and the passage number was similar amongst the different groups.

### Invasion Assay

Cell invasion assays were performed using BD BioCoat Matrigel Invasion Chambers (354480, BD Biosciences, San Jose, CA) per the manufacturer’s instructions. Briefly, 10,000 cells were plated inside the matrigel coated chambers in 500 μl of DMEM containing 2% BGS. 750 μl of DMEM containing 2% BGS was added to the outer chambers. Experimental and control groups were established with or without the supplementation of IL-10 (200 ng/ml) and/or 4-MU (0.3 mM/ml) to the inner chamber only. After 48 hours, non-invading cells were removed from interior surface of the membrane by scrubbing gently using a cotton-tipped swab. Each insert was then fixed and stained using Diff-Quik staining (NC9943455, Fisher Scientific, Waltham, MA) as per manufacturer’s instructions, with a two minute incubation in Diff-Quik fixative, solution I and solution II. The chambers were then washed in water; the membranes were gently cutout and mounted onto histology slides with Prolong Gold (Life Technologies, Carlsbad, CA) for imaging. Micrographs were taken of 10 fields at 40X magnification per sample (S3 Fig) using a Nikon 80i microscope and Nikon Elements Software, v3.2 (Nikon Instruments, Melville, NY). The number of cells in each field were counted and averaged over the 10 fields for each experimental group, with triplicates performed per treatment or control group with cells from three independent isolations (n = 3) and the passage number was similar amongst the different groups.

### Statistical Analysis

Statistical significance of the data was evaluated using analysis of variance (ANOVA), followed by post hoc tests (Tukey) and Student’s t-test when appropriate. MS-Excel (Microsoft, Redmond, WA) was used for the purpose.

## Results

### FFB have a distinct functional profile compared to AFB

**Fig 1:**
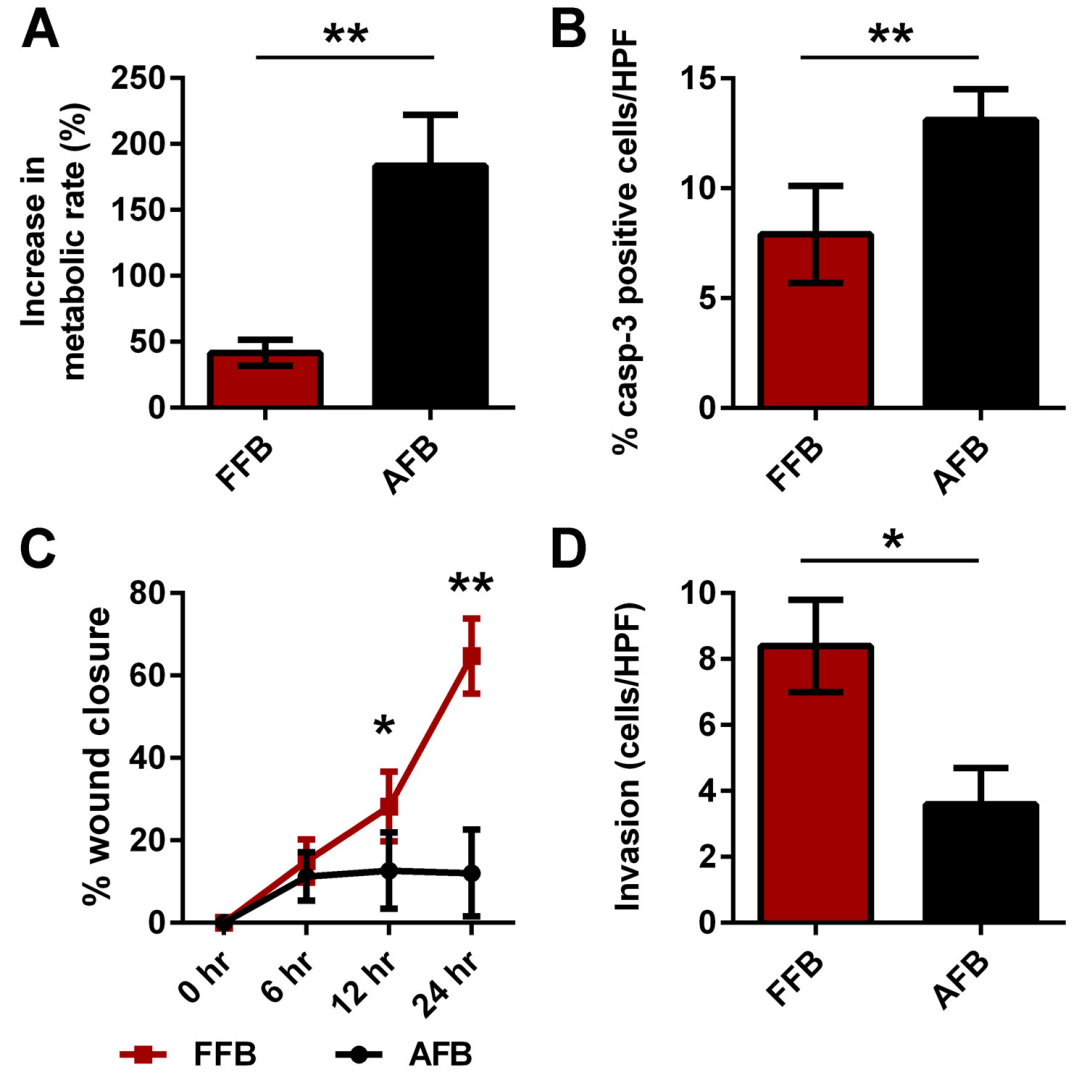
Differences in the functional profile of FFB compared to AFB. A) Rate of metabolism, B) apoptosis, C) migration and D) invasion were compared between FFB and AFB. Bar plots represent average±SD. Asterisks denote statistically significant differences between the groups (* p<0.05, ** p<0.01; Student’s t-test; n = 3 per group at similar passage number; each experiment was conducted in triplicates with cells from independent isolations).

To assess for functional differences between FFB and AFB, we compared the rate of metabolism, apoptosis, migration and invasion (Fig 1). Metabolic activity of the fibroblasts was assessed using an MTS assay. The increase in the metabolic rate compared to their baseline activity at 48 hours was significantly lower in the FFB compare to AFB (41.6±9.8% vs. 183.6±38.5%, p<0.01) (Fig 1A). Similarly, the rate of apoptosis, evaluated through immunostaining for caspase-3, demonstrated that FFB have a significantly decreased rate of apoptosis compared to AFB (7.9±2.2% vs.13.1±1.4%, p = 0.01) (Fig 1B). Next, a scratch wound assay was conducted to evaluate the differences in rate of fetal versus adult fibroblast migration by comparing the percent closure of the scratch defect at multiple time points. At 6 hours after creation of the scratch defect, the difference in the migration between FFB and AFB was not statistically significant (14.97±5.3% vs. 11.3±5.9, p = ns) (Fig 1C). However, by 12 hours, FFB demonstrated significantly increased migration compared to AFB (28.22+8.47% vs. 12.64 ± 9.23, p<0.05) (Fig 1C). FFB continued to demonstrate increased migration in comparison to AFB even at 24 hours (64.72±9.12% vs. 12.06±10.54%, p<0.01) (Fig 1C). Lastly, the difference in ability of FFB and AFB to invade through a matrix was evaluated using a matrigel invasion transwell assay. FFB demonstrated significantly more invasion compared to AFB (8.4±1.4 cells/HPF vs. 3.6±1.1 cells/HPF, p<0.05) (Fig 1D).

Together, these results indicate that FFB exhibit a more dynamic phenotype than AFB, characterized by greater migration and invasion, and less apoptosis than AFB. Notably, because FFB demonstrate less metabolic activity than AFB, it seems unlikely that the enhanced migration and invasion seen by AFB is merely a byproduct of greater activation. Also, the fact that these observations were made *in vitro* indicated that these attributes were intrinsic to the FFB themselves, rather than being predicated on their location within fetal tissues.

### IL-10 is essential to FFB migration and invasion

**Fig 2:**
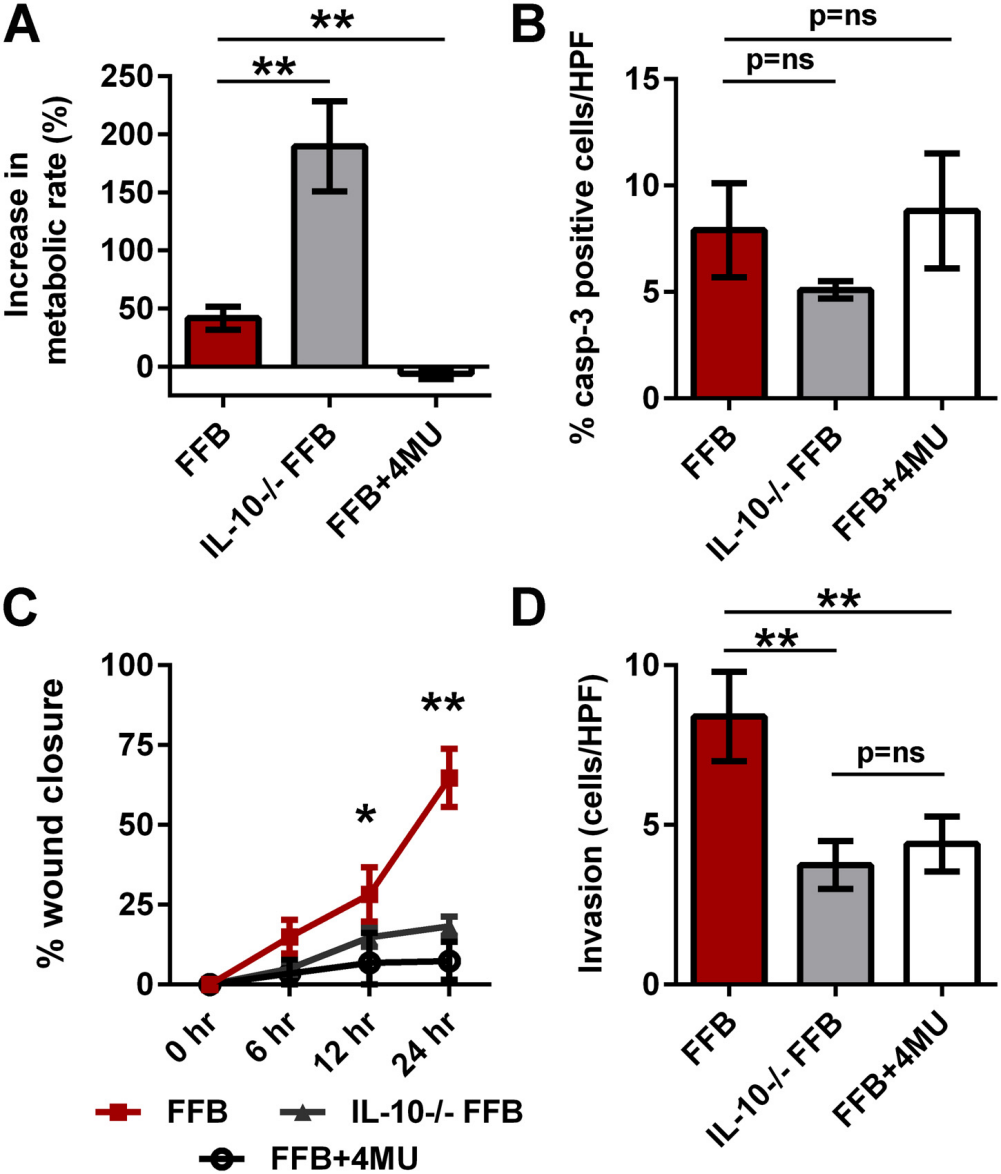
The role of IL-10 and hyaluronan in the functional profile of FFB. A) Rate of metabolism, B) apoptosis, C) migration and D) invasion were compared between FFB and IL-10 knockout FFB to determine if IL-10 is essential to the functional profile of the FFB, and between FFB and FFB in presence of 4-MU to determine if hyaluronan is essential to the functional profile of the FFB. Bar plots represent average±SD. Asterisks denote statistically significant differences between the groups (* p<0.05, ** p<0.01; Student’s t-test and ANOVA; n = 3 per group at similar passage number; each experiment was conducted in triplicates with cells from independent isolations).

The role of IL-10 in the fetal fibroblast functional profile was evaluated by comparing the differences in the rate of metabolism, apoptosis, migration and invasion between the control C57BL/6J FFB and transgenic *IL-10* ^*-/-*^ FFB of the same gestational age (E14.5). *IL-10* ^*-/-*^ FFB demonstrated a significant increase in metabolic rate at 48 hours compared to control FFB (41.6±9.8% vs. 189.63±38.8%, p<0.01) (Fig 2A). However, the *IL-10* ^*-/-*^ FFB had a similar rate of apoptosis to control FFB (7.9+2.2% vs. 5.1+0.4%, p = 0.17) (Fig 2B). Loss of IL-10 resulted in a significant decrease in migration to close a scratch wound defect in FFB at 12 and 24 hours, with FFB from control mice having greater “wound closure” rates than FFB from *IL-10* ^*-/-*^ mice (12 hours: 28.22+8.47% vs. 14.8+3.0%, p = 0.03; 24 hours: 64.72±9.12% vs. 18.24+3.0%, p<0.01) (Fig 2C). Control FFB also had significantly greater invasion capability compared to *IL-10* ^*-/-*^ FFB (8.4+1.4 cells/HPF vs. 3.7+0.7, p<0.01) (Fig 2D).

These data demonstrate that IL-10 is essential to the dynamic phenotype of FFB described in Fig 1. Consistent with this, our data indicate that *IL-10* ^*-/-*^ FFB have a phenotype similar to the AFB. The rate of metabolic activity, migration and invasion of the *IL-10* ^*-/-*^ FFB is not statistically different when compared to AFB (S4 Fig).

### HA synthesis is essential to FFB migration and invasion

We have previously established that *IL-10* ^*-/-*^ FFB produce significantly lower levels of HA and are characterized by reduced HA-pericellular matrix coats *in vitro* and reduced HA levels in cutaneous wounds *in vivo* [37, 38]. In addition, IL-10 supplementation led to increased HA production by AFB [36]. To evaluate the role of HA in the IL-10 mediated fetal functional phenotype described here, experiments were performed by competitively inhibiting HA synthase function using 4-MU. This drug was added to cell culture media at 0.3 mM/ml and the effect on metabolism, apoptosis, migration and invasion was determined. HA synthase inhibition resulted in a significantly decreased metabolic activity at 48 hours compared to normal control FFB (6.3±4.6% vs. +41.6±9.8%, p<0.01) (Fig 2A). Competitive inhibition of HA synthases has no effect on apoptosis of FFB when compared to normal FFB (7.9+2.2% vs. 8.8+2.7%, p = 0.64) (Fig 2B). However, compared to control FFB, HA synthase inhibition resulted in a significant decrease in migration of FFB to close a scratch wound defect at 12 and 24 hours (@ 12 hours: 28.22+8.47% vs. 6.8+9.3%, p<0.01; @ 24 hours: 64.72±9.12% vs. 7.43+5.9%, p<0.01) (Fig 2C), as well as a significant decrease in invasion capability (8.4+1.4 cells/HPF vs. 4.4+0.9, p<0.01) (Fig 2D).

These data indicate that inhibition of HA synthesis leads to a loss of the enhanced functional profile associated with FFB *in vitro*. Of note, HA synthase inhibition in normal FFB resulted in a migration and invasion rates similar to IL-10^*-/-*^ FFB at the same gestation age (Fig 2C and 2D).

### IL-10 recapitulates the fetal migration and invasion phenotype in AFB

**Fig 3:**
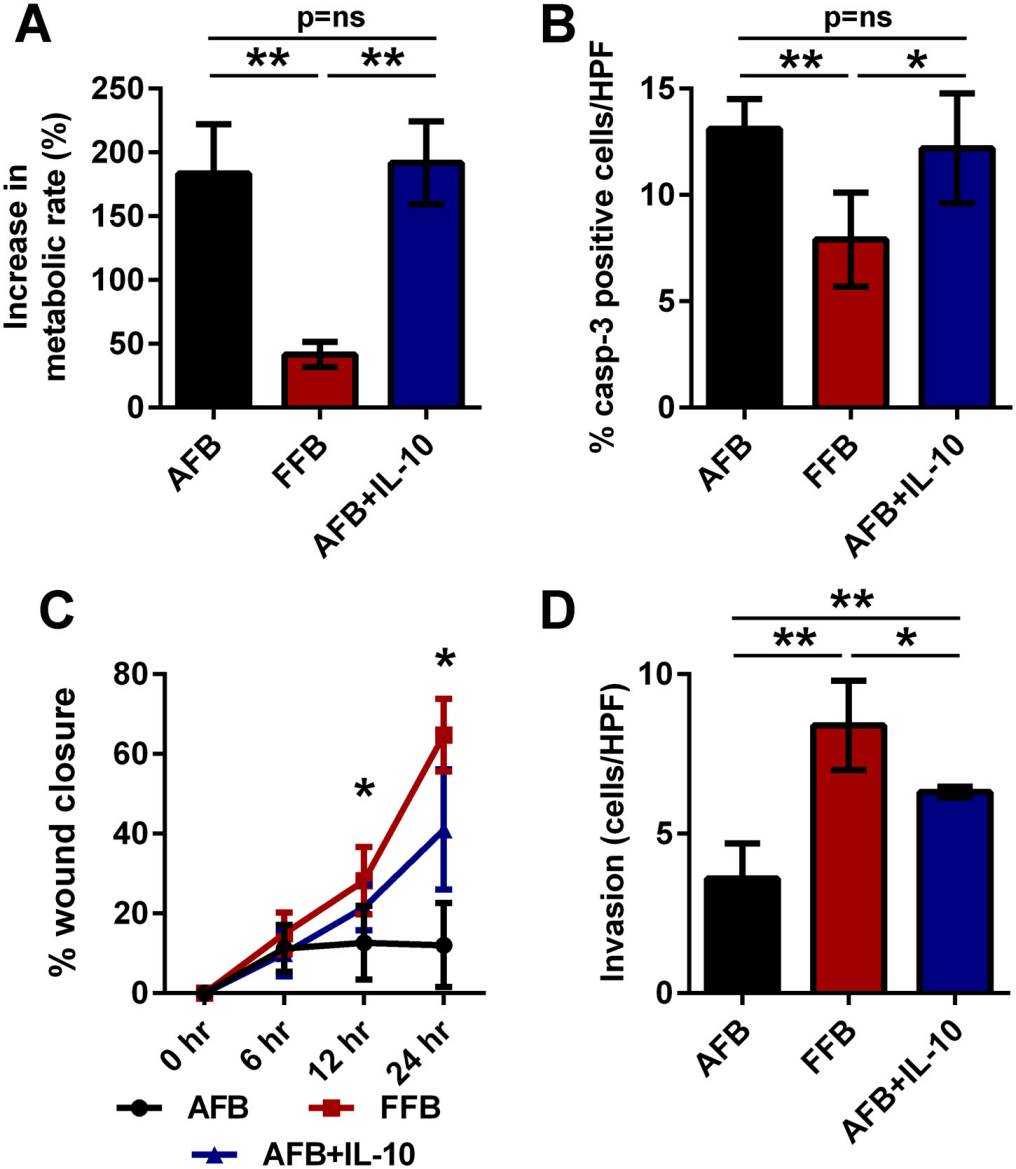
The role of IL-10 in recapitulating the fetal functional profile of AFB. A) Rate of metabolism, B) apoptosis, C) migration and D) invasion were compared between AFB, AFB+IL-10, and FFB to determine if IL-10 treatment can recapitulate the fetal functional profile in the FFB. IL-10 treatment recapitulated the migration and invasion aspects of the fetal-type fibroblast phenotype in AFB, but metabolism and apoptosis rates were not impacted by IL-10. Bar plots represent average±SD. Asterisks denote statistically significant differences between the groups (* p<0.05, ** p<0.01; Student’s t-test and ANOVA; n = 3 per group at similar passage number; each experiment was conducted in triplicates with cells from independent isolations).

Our data suggest that the dynamic phenotype associated with FFB was both HA and IL-10 dependent. Therefore, we next sought to evaluate the ability of IL-10 to recapitulate the fetal phenotype in AFB. To this end, experiments were performed where AFB were treated with recombinant murine IL-10, and similar functional parameters were evaluated. We found that the metabolic activity of AFB was unchanged with or without IL-10 supplementation (191.8±32.5% vs. 183.6±38.5%, p = ns) (Fig 3A), and that the increase in metabolic activity of the IL-10 treated AFB remained significantly higher than that of the FFB at 48 hours (191.8±32.5% vs. 41.6±9.8%, p<0.01) (Fig 3A). Similarly, we found that the rate of apoptosis of AFB was unchanged with or without IL-10 supplementation (12.2+2.6% vs. 13.1+1.4% (p = ns) (Fig 3B), and that the IL-10 treated AFB evidenced significantly increased apoptosis than the FFB (12.2+2.6% vs. 7.9±2.2%, p<0.05) (Fig 3B). However, IL-10 treatment significantly enhanced migration of the AFB as compared to untreated AFB (@ 12 hours: 21.5+5.7% vs. 12.64±9.23% (p<0.05); @ 24 hours: 41.04+15.07% vs. 12.06±10.54% (p<0.05)) (Fig 3C). The rate of migration of IL-10 treated AFB was comparable to FFB migration at 12 and 24 hours (@ 12 hours: 21.5+5.7% vs. 28.22+8.47% (p = ns); @ 24 hours: 41.04+15.07% vs. 28.22+8.47% (p = ns)) (Fig 3C). IL-10 treatment also enhanced invasion of the AFB as compared to the untreated AFB controls (6.3±0.2 cells/HPF vs. 3.6±1.1, p<0.01) (Fig 3D). Indeed, the invasion of IL-10 treated AFB was brought to levels comparable to the FFB invasion phenotype (6.3±0.2 cells/HPF vs. 8.4+1.4, p<0.05) (Fig 3D).

These data suggest that IL-10 treatment recapitulates aspects of the fetal—type fibroblast phenotype in AFB, in terms of migration and invasion, but that other aspects, metabolism and apoptosis rates were independent of IL-10.

### IL-10 mediated recapitulation of fetal functional properties in AFB is HA dependent

**Fig 4:**
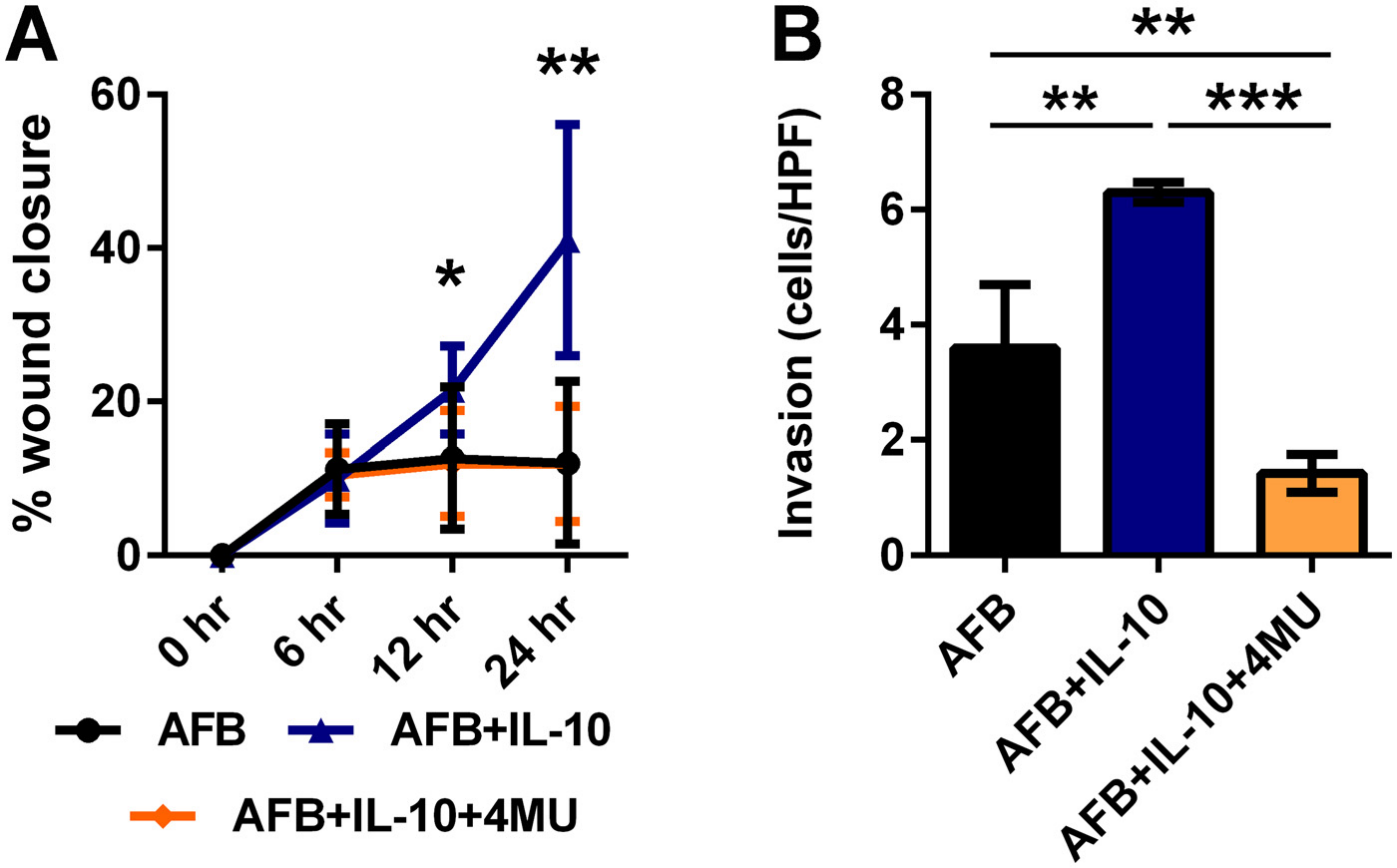
The essential role of HA in IL-10 mediated recapitulation of the fetal-type migration and invasion profile in AFB. A) migration and B) invasion were compared between AFB, AFB+IL-10, and AFB+IL-10 in the presence of 4-MU to determine if IL-10 mediated recapitulation of fetal-type fibroblast migration and invasion in AFB is HA dependent. The increase in migration and invasion with IL-10 supplementation was abrogated with competitive inhibition of HA synthases in AFB. Bar plots represent average±SD. Asterisks denote statistically significant differences between the groups (* p<0.05, ** p<0.01, *** p<0.001; Student’s t-test and ANOVA; n = 3 per group at similar passage number; each experiment was conducted in triplicates with cells from independent isolations).

To evaluate the contribution of HA to the IL-10 mediated recapitulation of fetal type migration and invasion in AFB, we inhibited HA synthase activity using 4-MU. We observed that competitive inhibition of HA synthases negated the increased migration produced by the addition of IL-10 to AFB at both 12 and 24 hours after the creation of the scratch wound defect (@ 12 hours: 11.98+6.9% vs. 21.51±5.7, p<0.05; @ 24 hours: 11.90±7.5% vs. 41.04+15.07%, p<0.01) (Fig 4A). Similarly, the increase in invasion with IL-10 supplementation was abrogated with competitive inhibition of HA synthases in AFB (1.4+0.3 vs. 6.3+0.2, p<0.001) (Fig 4B).

## Discussion

We report here the contribution of IL-10 mediated HA production to the fetal phenotype of FFB. Our work has two major new findings. First, our data supports the hypothesis that the FFB have a distinct functional profile from AFB and that these phenotypic differences are cell-intrinsic and independent of the fetal tissue environment, because they are evident *in vitro*. FFB demonstrate decreased rates of metabolic activity and apoptosis and increased rates of cellular migration and invasion compared to AFB. The properties of metabolic activity and apoptosis are independent of IL-10 and HA. In contrast, the properties of enhanced migration and invasion of the FFB are dependent on IL-10 and HA. The differences in the fetal and adult fibroblast functional profile may represent an evolutionary strategy to adapt to the environment [51]. AFB demonstrated higher rates of metabolic activity and apoptosis, and have been previously demonstrated to have increased inflammatory response when stimulated, compared to FFB [8, 9]. This may represent an increase in the cellular turnover rate that is in response to a more challenging and active environmental milieu in adult wounds compared to the protected environment of the fetus. This difference may serve to achieve wound closure at the expense of scar formation and reduced wound strength. Conversely, FFB have enhanced migratory properties which may allow the deposition of a more organized extracellular matrix [4], and with a previously accepted attenuated inflammatory milieu of the fetus, may lead to decreased scar formation and regenerative healing.

**Fig 5:**
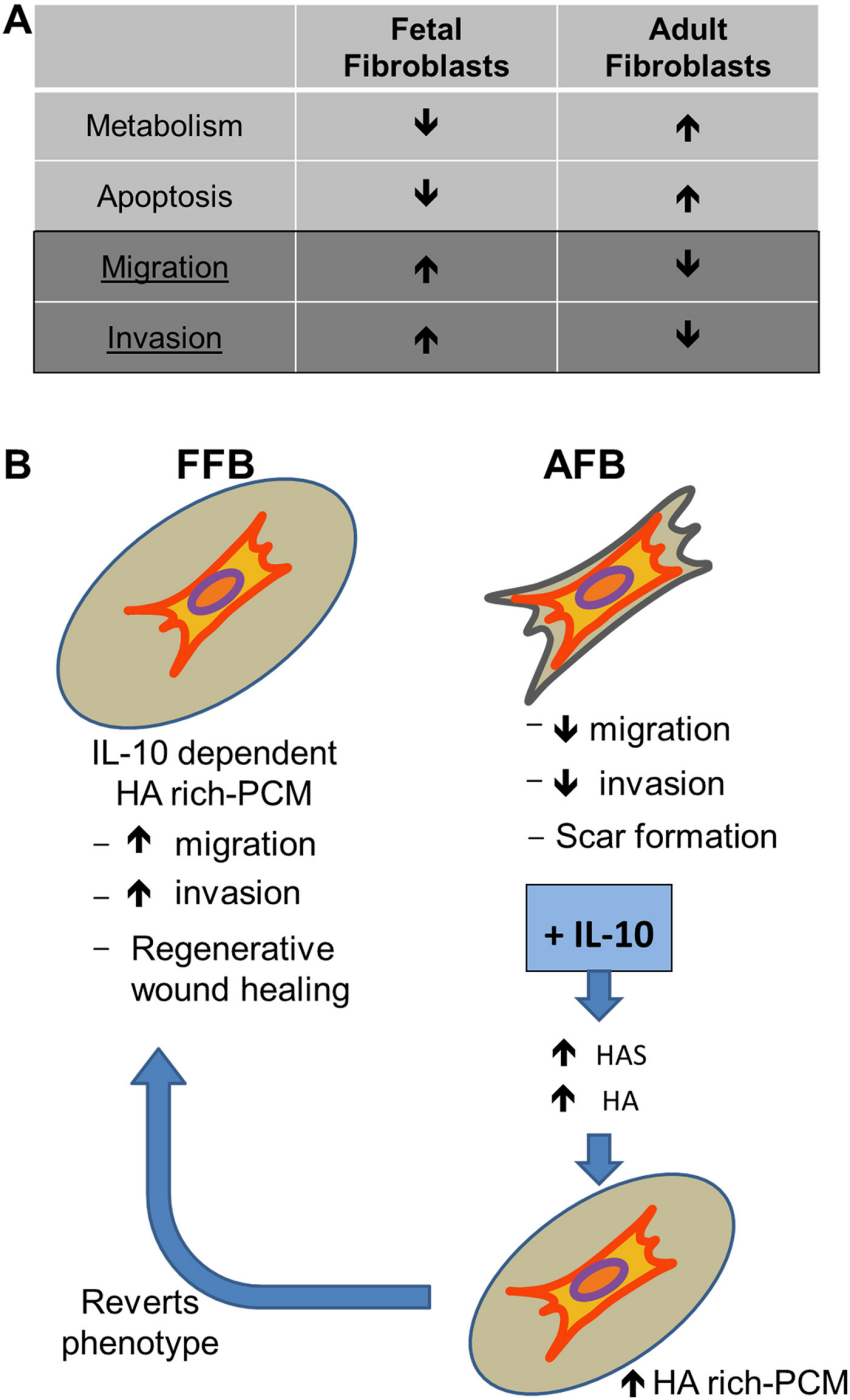
FFB have a distinct functional profile from AFB. A) FFB demonstrates decreased rates of metabolism and apoptosis and increased rates of cellular migration and invasion compared to AFB. IL-10 treatment can recapitulate the fetal functional phenotype of enhanced migration and invasion in AFB. B) Mechanistically, our previous data has shown that fetal fibroblasts synthesize more HA and HA-rich pericellular matrices (PCM) compared to AFB. IL-10 in the fetus has a novel role in the regulation of the HA-rich ECM synthesis. This is the first report that demonstrates that IL-10 mediated HA synthesis regulates the migration and invasion of the FFB, we posit this regulation, in part, facilitates the fetal regenerative response. In comparison to this, AFBs produce less HA and PCM and contribute to scar formation in postnatal wounds. IL-10 treatment increases HA synthesis and HA-rich PCM in AFB and revert them to a more fetal-type dynamic phenotype with increased migration and invasion, and recapitulate fetal phenotype in postnatal wounds.

Second, these data demonstrate that IL-10 treatment can recapitulate the fetal functional phenotype of enhanced migration and invasion in AFB. There are no prior data that demonstrate that IL-10 has an effect on fibroblast motility. Our data suggest that this may occur through an HA synthase-dependent mechanism, which is supported by the abrogation of IL-10’s positive effects in the presence of the HA synthase inhibitor 4-MU (Fig 5). These data build on our previous work showing that IL-10, a potent anti-inflammatory cytokine produced by multiple immune cells early in wound healing [52], plays a critical role in fetal regenerative wound healing [31, 32]. Mechanistically, it has been shown that IL-10 in the fetus not only regulates the inflammatory response [32] but also has a novel role in the regulation of the HA-rich ECM synthesis, suggesting pleiotropic roles of IL-10 in regenerative healing. Furthermore, we and others have previously demonstrated that overexpression of IL-10 in postnatal wounds creates an environment permissive of regenerative healing, which was attributed primarily to the known anti-inflammatory properties of IL-10. Recently, we also have substantial evidence that demonstrates a largely unknown role of IL-10 treatment in the regulation of the HA-rich ECM in postnatal wounds [36–38]. The current study extends this previous work by bringing it to the level of IL-10 and HA effects on specific FFB functions: namely migration and invasion. Moreover, we have demonstrated a novel-role for IL-10 as a regulator of the fetal fibroblast functional profile which is dependent on HA synthesis. These data may account at least in part for the mechanism of how the fetus heals without scar and IL-10’s ability to achieve regenerative healing in postnatal wounds.

Our work implicates a major role for HA in fetal wound healing. It has been well described that the fetus is characterized by elevated and prolonged levels of HA which plays a crucial role in fetal tissue repair [53]. Consistent with this, Caskey RC *et al*. reported similar findings of decreased inflammatory response and regenerative healing in murine cutaneous wounds treated with lentiviral overexpression of HAS-1 [54]. The large, flexible, hydrophilic molecules of HA also have the ability to retain water and disperse solutes, and may provide an environment for fibroblasts to efficiently course through the wound. This in part explains the better migratory properties of FFB compared to the AFB as observed in the current study. The ability of FFB to migrate more efficiently compared to AFB has been correlated with fetal ability to synthesize HA [30]. The role of HA in cell migration is further established by the fact that it has also been to shown to potentiate migration in various other cells types, including neural crest cells and cardiac cushion cells [55, 56]. While the mid-gestational fetal skin is rich in IL-10 and HA, as the development progresses, both IL-10 and HA levels decline [57, 58], which is commensurate with the onset of a scarring phenotype in the third trimester.

In order to facilitate effective wound healing, fibroblasts must migrate and invade through various environments, including fibrin clot, the provisional matrix during the proliferative phase and a more collagenous matrix during remodeling [59]. The initial wave of fibroblasts migrate into the wound bed in a directed fashion in response to chemokines and growth factors produced by inflammatory cells, which then proceed to modulate keratinocyte migration and re-epithelialization through paracrine signaling [60]. In post-natal wounding, these fibroblasts differentiate into myofibroblasts, characterized by the expression of alpha-smooth muscle actin, and are capable of exerting contractile forces in the wound. These myofibroblasts are notably absent in fetal wounds that heal without scar [61] and have been shown to have distinct characteristics when compared to undifferentiated post-natal fibroblasts. For instance, myofibroblasts have been demonstrated to interact differently with keratinocytes compared to fibroblasts with a unique pattern of cytokine secretion. Further study is necessary to characterize the effect of IL-10 and HA on myofibroblasts.

Our results suggest that IL-10 mediated HA synthesis is essential to the fetal fibroblast functional profile which can be recapitulated in AFB by addition of IL-10. The precise relation between IL-10 and HA molecules has not been completely elucidated. It has been shown that wounds in IL-10^*-/-*^ adult mice close significantly faster as compared to wild type controls, albeit with exaggerated scarring and impaired wound strength [62], while wound treatment with HMW-HA increases time to completely heal, but results in better tissue repair and restoration of the repair tissue integrity [63]. Targeted manipulations of IL-10 and HA signaling within fibroblasts may represent new therapeutic targets to achieve improved wound healing and reduce scar formation.

## Conclusions

This study reports the differences between functional profile of the FFB and AFB, some of which may be crucial for the regenerative wound healing observed in fetal skin. To the best of our knowledge, this is the first report that demonstrates that IL-10 mediated HA synthesis regulates the migration and invasion of the FFB and that IL-10 treatment can recapitulate this functional phenotype in postnatal fibroblasts. This functional role of IL-10 may be essential to the fetal regenerative phenotype. While these studies have been confined to the skin, understanding the fetal regenerative response to injury may have therapeutic implications for any pathology characterized by excessive fibroplasia.

## Supporting Information

S1 Fig Baseline viability curves for different cell types. Cellular metabolic activity was determined using an MTS assay. To account for the cellular differences between the different cell types studied, a respective baseline viability curve was developed for each cell type in question.
(TIF)

S2 Fig Quantification of cell migration using a scratch wound closure assay. Cells were plated on 12-well cell culture plates. A scratch defect was created in the cell monolayer along the diameter. Four points were marked along the scratch defect as reference points to capture photographic images to trace defect closure at multiple time points. Representative 4X images from each group are shown. The lines represent the scratch defect edges.
(TIF)

S3 Fig Quantification of cell invasion using a matrigel invasion assay. Cells were plated on a transwell porous membrane coated with matrigel. The cells that invaded through the matrigel matrix and passed the porous membrane to the outer side were identified by Diff-quik staining. Representative high-power (40X) images from stained membranes from each treatment group are shown. Arrows represent the pores in the membranes.
(TIF)

S4 Fig IL-10 is essential to the dynamic phenotype of FFB described in Fig 1. *IL-10* ^*-/-*^ FFB have a phenotype similar to the AFB. The rate of metabolic activity (A), migration (C) and invasion (D) of the IL-10-/- FFB is not statistically different when compared to AFB. Bar plots represent average±SD. Asterisks denote statistically significant differences between the groups (** p<0.01; Student’s t-test; n = 3 per group at similar passage number; each experiment was conducted in triplicates with cells from independent isolations).
(TIF)
