# Supplementary figures and images for: The Role of Interleukin-10 and Hyaluronan in Murine Fetal Fibroblast Function In Vitro: Implications for Recapitulating Fetal Regenerative Wound Healing
Source: PLoS One. 2015 May 7;10(5):e0124302. doi: 10.1371/journal.pone.0124302 (PMC4423847; doi:10.1371/journal.pone.0124302)

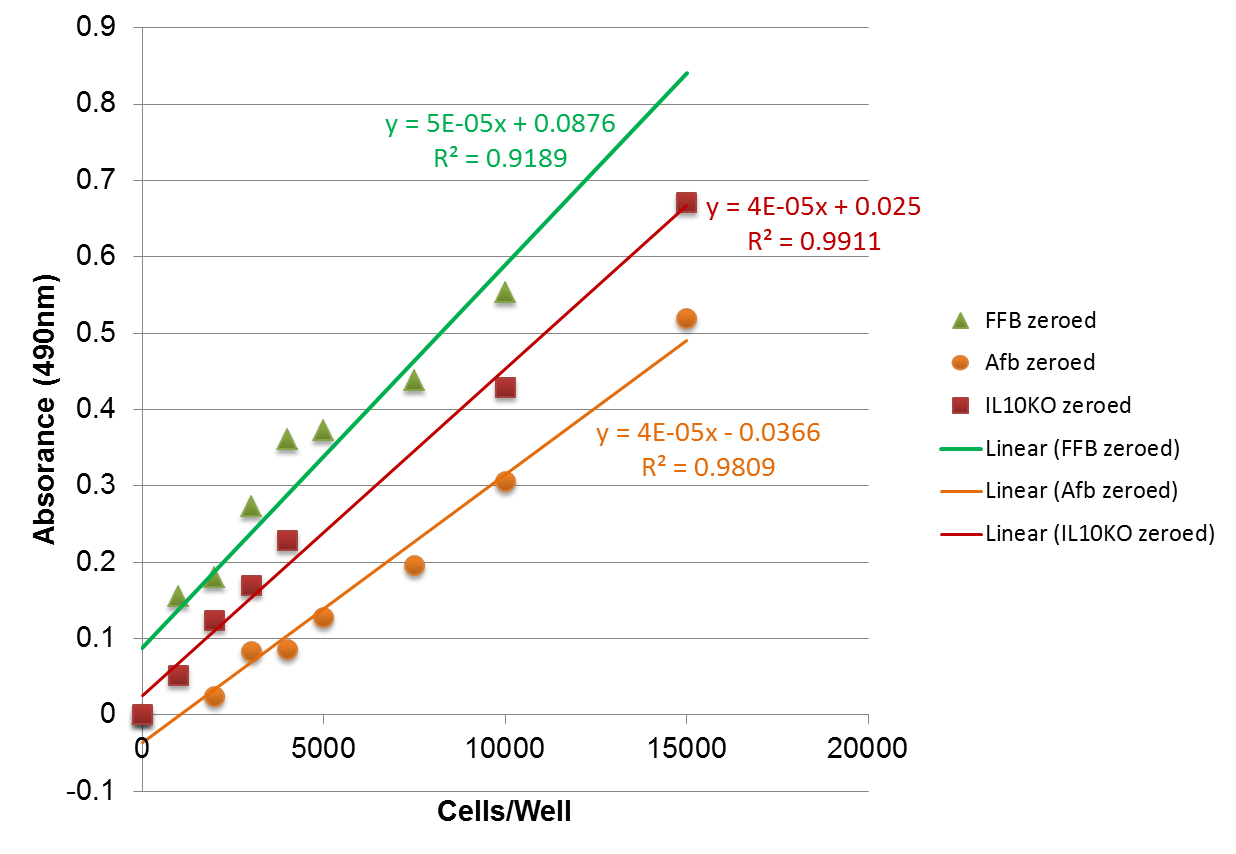

Supplement: S1 Fig — Cellular metabolic activity was determined using an MTS assay. To account for the cellular differences between the different cell types studied, a respective baseline viability curve was developed for each cell type in question. (TIF) [file pone.0124302.s001.tif]

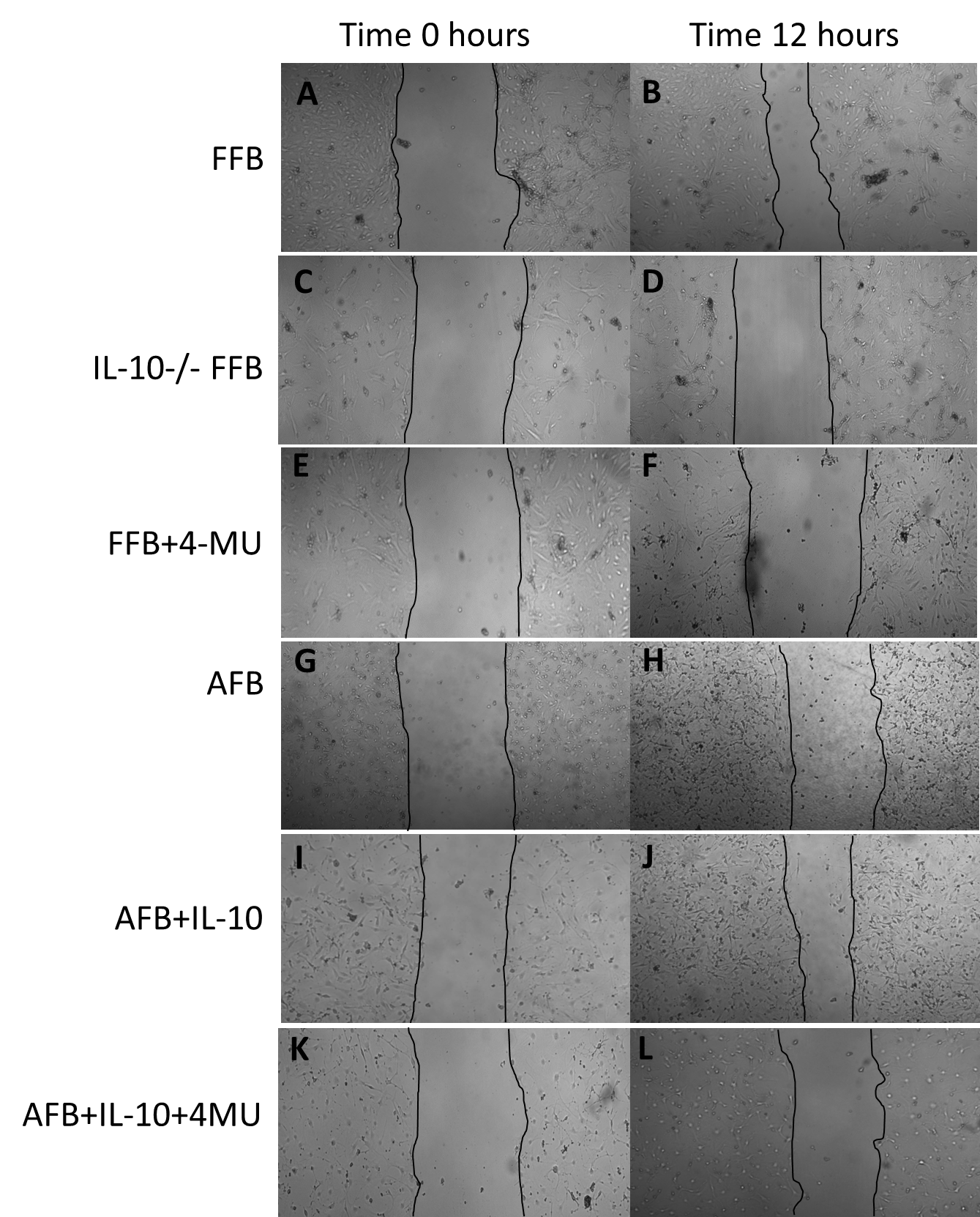

Supplement: S2 Fig — Cells were plated on 12-well cell culture plates. A scratch defect was created in the cell monolayer along the diameter. Four points were marked along the scratch defect as reference points to capture photographic images to trace defect closure at multiple time points. Representative 4X images from each group are shown. The lines represent the scratch defect edges. (TIF) [file pone.0124302.s002.tif]

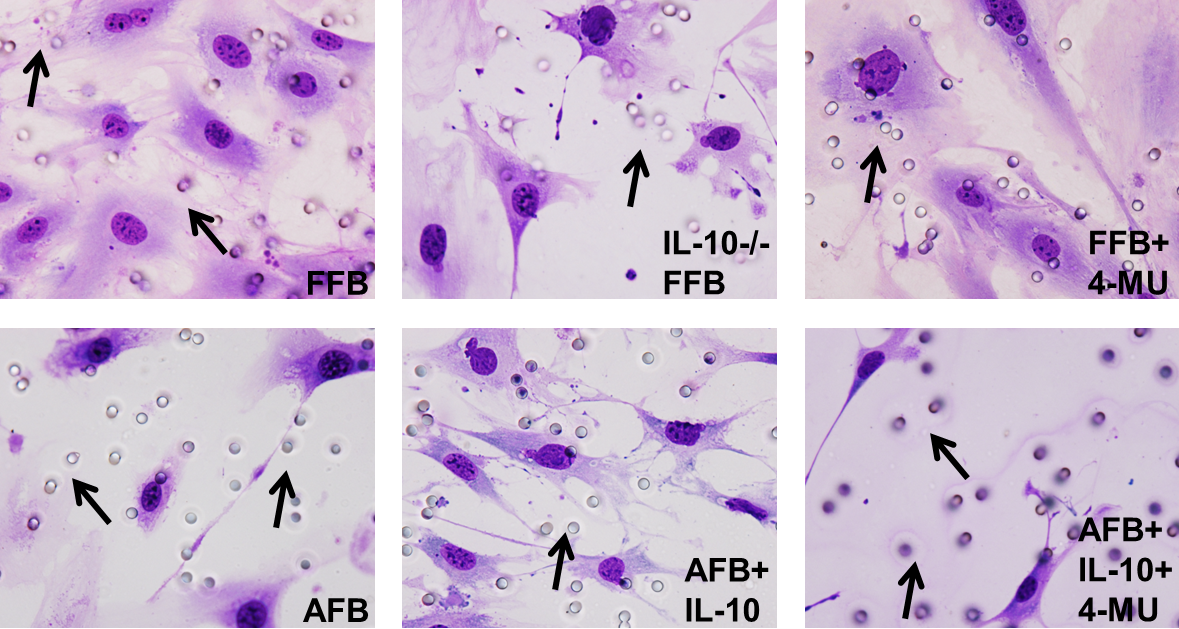

Supplement: S3 Fig — Cells were plated on a transwell porous membrane coated with matrigel. The cells that invaded through the matrigel matrix and passed the porous membrane to the outer side were identified by Diff-quik staining. Representative high-power (40X) images from stained membranes from each treatment group are shown. Arrows represent the pores in the membranes. (TIF) [file pone.0124302.s003.tif]

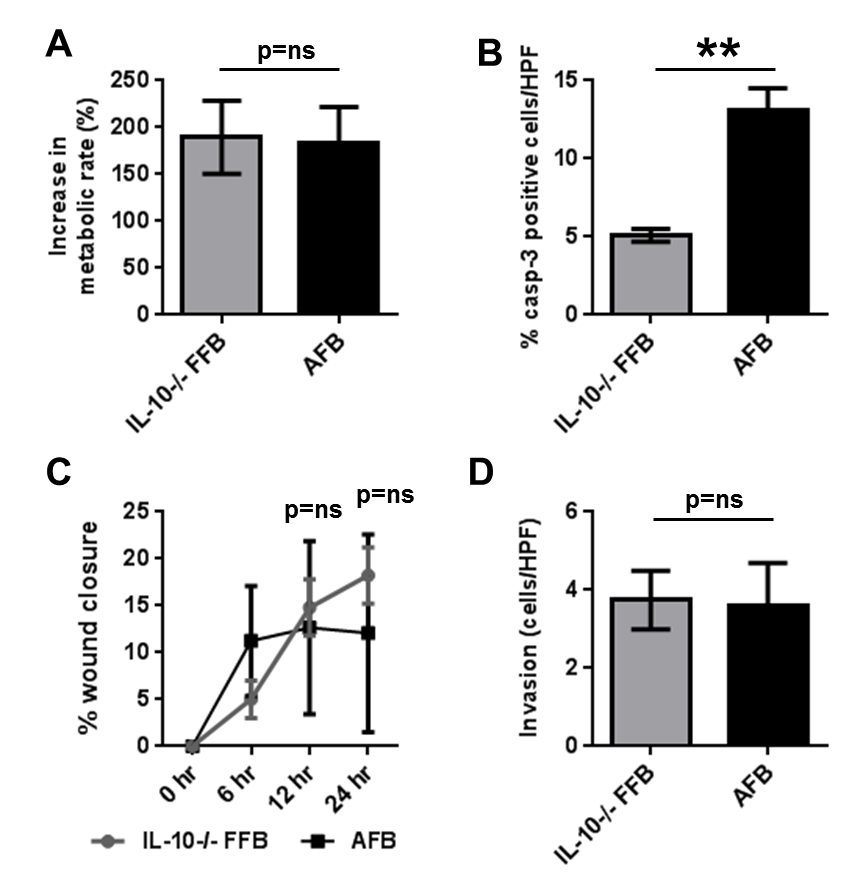

Supplement: S4 Fig — IL-10 -/- FFB have a phenotype similar to the AFB. The rate of metabolic activity (A), migration (C) and invasion (D) of the IL-10-/- FFB is not statistically different when compared to AFB. Bar plots represent average±SD. Asterisks denote statistically significant differences between the groups (** p<0.01; Student’s t-test; n = 3 per group at similar passage number; each experiment was conducted in triplicates with cells from independent isolations). (TIF) [file pone.0124302.s004.tif]
